# Potential application of the oxidative nucleic acid damage biomarkers in detection of diseases

**DOI:** 10.18632/oncotarget.20801

**Published:** 2017-09-08

**Authors:** Cheng Guo, Peili Ding, Cong Xie, Chenyang Ye, Minfeng Ye, Chi Pan, Xiaoji Cao, Suzhan Zhang, Shu Zheng

**Affiliations:** ^1^ Cancer Institute, Key Laboratory of Cancer Prevention and Intervention, China National Ministry of Education, The Second Affiliated Hospital, Zhejiang University School of Medicine, Hangzhou, Zhejiang 310009, China; ^2^ College of Chemical Engineering, Zhejiang University of Technology, Hangzhou, Zhejiang 310014, China; ^3^ Department of Gastrointestinal Surgery, Shaoxing People’s Hospital, Shaoxing Hospital of Zhejiang University, Shaoxing, Zhejiang 312000, China; ^4^ Research Center for Air Pollution and Health, Zhejiang University, Hangzhou, Zhejiang 310009, China

**Keywords:** oxidative DNA damage, oxidative RNA damage, ROS, biomarker, cancer

## Abstract

Reactive oxygen species (ROS) are generated after exposure to harmful environmental factors and during normal cellular metabolic processes. The balance of the generating and scavenging of ROS plays a significant role in living cells. The accumulation of ROS will lead to oxidative damage to biomolecules including nucleic acid. Although many types of oxidative nucleic acid damage products have been identified, 8-oxo-7,8-dihydro-2’-deoxyguanosine (8-oxodG) and 8-oxo-7,8-dihydroguanosine (8-oxoG) has been commonly chosen as the biomarkers of oxidative damage to DNA and RNA, respectively. It has been demonstrated that oxidative damage to nucleic acid is an initiator in pathogenesis of numerous diseases. Thus, oxidative nucleic acid damage biomarkers have the potential to be utilized for detection of diseases. Herein, we reviewed the relationship of oxidative nucleic acid damage and development of various diseases including cancers (colorectal cancer, gastrointestinal cancer, breast cancer, lung cancer, epithelial ovarian carcinoma, esophageal squamous cell carcinoma), neurodegenerative disorders and chronic diseases (diabetes and its complications, cardiovascular diseases). The potential of oxidative nucleic acid damage biomarkers for detection of diseases and drug development were described. Moreover, the approaches for detection of these biomarkers were also summarized.

## INTRODUCTION

Reactive oxygen species (ROS) are generated continuously during physical and other metabolic reactions in living cells of organism. Superoxide anion, hydroxyl radical, hydrogen peroxide and singlet oxygen are well-known ROS [1]. The endogenous ROS also have important regulatory effect in human signal transduction pathways [2]. Keeping the balance between clearance and generation of ROS plays a critical role in living cells. In human resting state, the antioxidant system of body maintains the redox equilibrium which can remove excess free radicals timely. The antioxidant systems include simple antioxidants such as vitamin C and E, which can intercept free radicals and prevent cellular biomolecules from damage. Other antioxidant systems are enzymatic systems including superoxide dismutase (SOD), catalase, glutathione peroxidase and others [3]. All these systems possess different abilities to limit the concentration of unusual accumulated ROS. When organism is in pathological state or exposed to other precarious exogenous factors, increasing ROS accumulated *in vivo*, without be removed timely, would lead to a redox imbalance. Its reactive nature can cause extensive oxidative damage to nucleic acid, lipids, and other important cellular structures, leading to cell dysfunctions, metabolic disorders, or even mutations, causing numerous diseases at the end of time [4].

**Figure 1:**
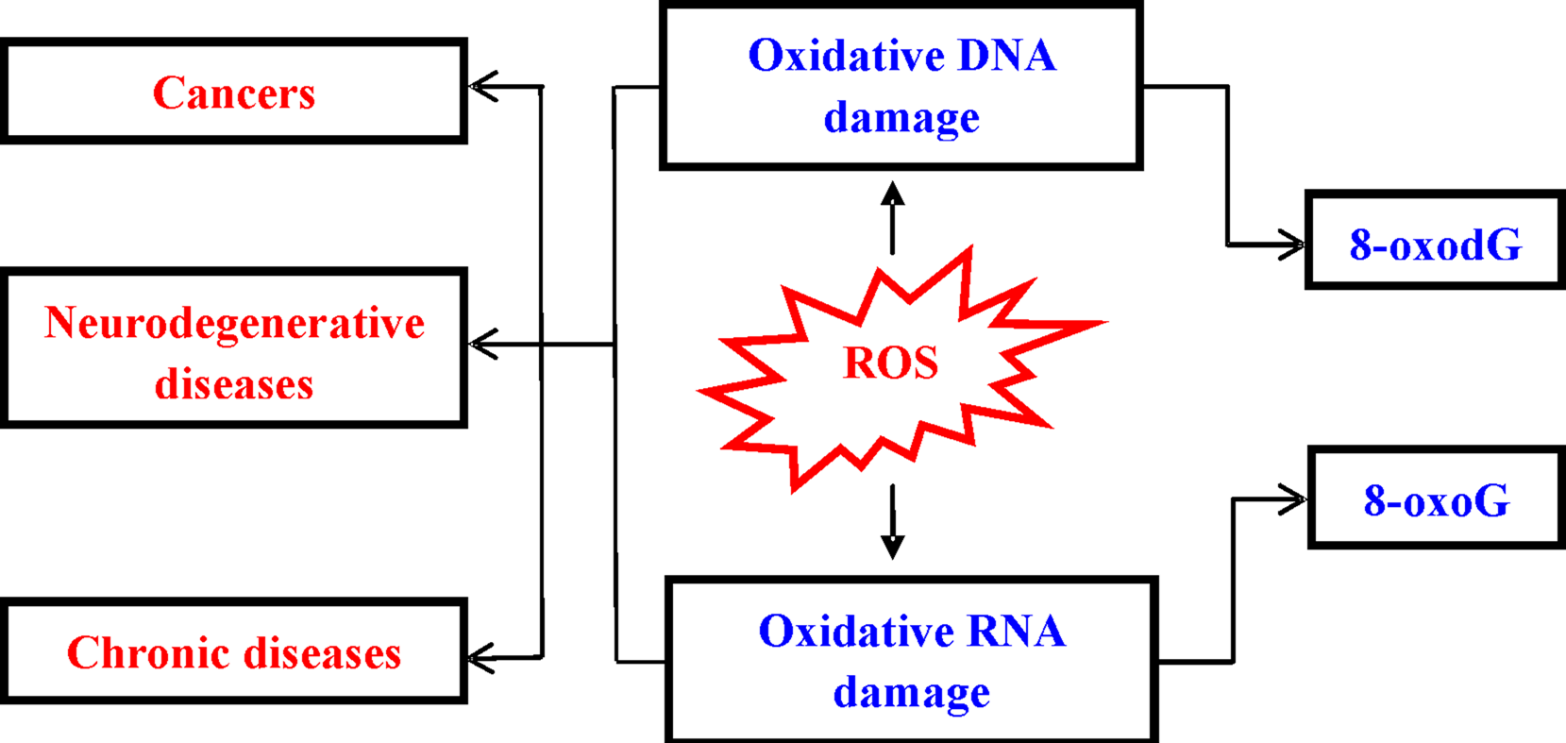
Consequence of ROS-induced nucleic acids damage

Oxidative damage to nucleic acid can cause base substitution, addition, deletion, and other mutations. During the process of oxidative damage, many nucleic acids oxidized products are generated. 8-Oxo-7,8-dihydro-2’-deoxyguanosine (8-oxodG) and 8-oxo-7,8-dihydroguanosine (8-oxoG) are predominant ROS-induced oxidative modifiers among different types of oxidative products [5–6]. Herein, we discussed the relationship between oxidative nucleic acid damage and development of various diseases (Figure 1). Then, we described the potential of oxidized biomarkers in diseases detection and drug development. Finally, the approaches for detection of these biomarkers were summarized.

## OXIDATIVE NUCLEIC ACIDS DAMAGE

Hydroxyl radical, as one of the most important reactive oxygen species, plays a vital role in the stress reaction causing damage to nucleic acids and other biomolecules. It can be generated by different mechanisms, for example, undecomposed hydroxyl peroxide parts turning into hydroxyl radical through Fenton reaction (Fe^2+^ + H_2_O_2_→Fe^3+^ + ∙OH + OH^-^) [7]. If accumulated ROS is not scavenged opportunely, redox balance will break up, resulting in the dysfunction of cellular biological detoxification and repair mechanism [8]. Excessive hydroxyl radical attacks adjacent DNA strands which exist not only in cellular but also in mitochondrial, eventually leading to the creation of all kinds of oxidation products [9]. RNA is nearly single strand, and thus the bases can not be protected by hydrogen bonding. In addition, there is also lack of specific RNA protected protein in living cells [10]. Therefore, RNA bases are more susceptible to oxidative damage and oxidative damage to RNA is also accused to hydroxyl radical with the feature of high reactivity and difficult diffusion [11–12]. However, compared with oxidative DNA damage, less effort has been devoted to the investigation of oxidative RNA damage.

**Figure 2:**
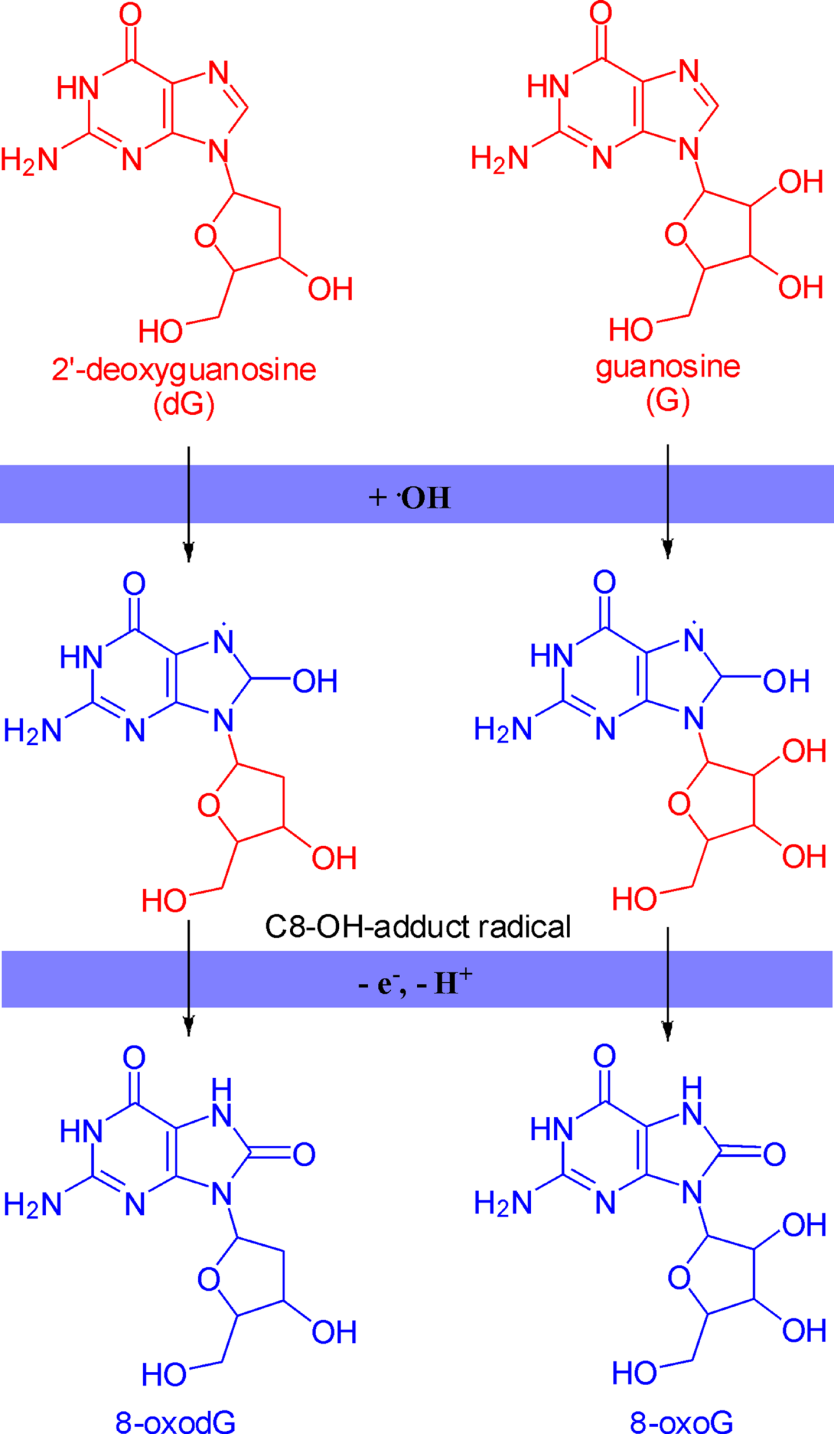
The formation mechanism of 8-oxodG and 8-oxoG

The major product of the reaction of 2’-deoxyguanosine or guanosine with hydroxyl radical is C8-OH-adduct radical. Then C8-OH-adduct radical loses an electron and a proton to form 8-oxodG or 8-oxoG (Figure 2) [1, 13]. 8-OxodG/8-oxoG is the most abundant oxidized products because it is stable and relatively easily formed, though there are other bases reacting with hydroxyl radical in a similar way [6, 14].

8-OxodG is cut off from the DNA chain under the effect of a variety of self-protection mechanisms, e.g., base excision repair, mismatch repair and nucleotide excision repair, and then excreted into urine without further metabolism. As a metabolic end product, the level of 8-oxodG is maintained at stable level *in vivo* fluid and not affected by factors such as diet [15]. However, the mechanism of RNA repair is still not clear and deserved further investigation.

The half-life period of 8-oxodG is longer than other oxidized products, and it can be easily detected in clinical practice [16, 17]. 8-OxodG mainly induces the transversion from GC to TA, GC to AT, or GC to CG, and this is a vital mechanism of ROS induced mutagenesis [14]. Oxidative damage also affected normal function of RNA, including ribosome dysfunction, low translation efficiency and protein synthesis disorders (declining protein synthesis rates, production of aggregated peptides), causing dysregulation of gene expression and errors in key proteins that involved in the chronic-degenerative diseases especially neurodegenerations [10, 11, 18]. Carcinogenesis is a long evolution process of multistage, including DNA mutation, activation of oncogenes and inactivation of tumor suppressor genes, changes of apoptosis regulating genes and DNA repair genes. Mutation is one of the most serious consequences of oxidative DNA damage and it is a critical step in carcinogenesis [19].

## POTENTIAL APPLICATION IN DETECTION OF DISEASES

In previous studies, oxidative nucleic acids biomarkers were considered to be involved in the occurrence and development of different types of cancers, neurodegenerative disorders and chronic diseases (Supplementary Table 1). As shown in this table, oxidative damage exists in various diseases, and the biomarkers are over-expressed in patients compared with controls. Concentrations of oxidative damage biomarkers in patients with diseases are about 1.3 to 5-fold higher than that in controls. Although there may have some discrepancy in a few studies. This implies that 8-oxodG/8-oxoG might be a good indicator of diseases and has potential to be a useful clinical biomarker to warn or diagnosis some relevant diseases in the future.

### Oxidative damage biomarkers and different cancers

#### Colorectal cancer

As we all know, excessive accumulation of ROS *in vivo* can cause chronic enteritis, increasing the risk of colorectal cancer especially oxygen-radical-mediated inflammatory colorectal cancer. In organism, many endogenous antioxidant enzymes can defense ROS-mediated damage, maintaining the balance of oxidant-antioxidant system. 8-OxodG concentrations, glutathione peroxidase, and superoxide dismutase activity sharply increased in plasma from patients with inflammatory bowel disease (IBD) [20]. This may help us to understand why there is a significantly increased risk of colorectal cancer occurrence for patients with IBM, compared to non-IBM. Chang and his colleagues found serum 8-oxodG concentrations highly increased in colorectal cancer patients compared to the control group (*p* < 0.01) [21]. Sato *et al.* measured plasma levels of 8-oxodG from 58 with benign tumor, 32 with early colorectal cancer, 25 with advanced colorectal cancer, 36 healthy controls, and they found that 8-oxodG levels were highest in patients with early cancer [22]. Therefore, we suspect that 8-oxodG could be a potential biomarker for warning colorectal cancer in early stage.

Urine, compared with other biofluids such as plasma and serum, was considered to be a preferred biological matrix in clinical practice since it could be easily obtained in large volumes and it was noninvasive to patients. Recently, we have launched a study involved 142 healthy volunteers and 84 colorectal cancer patients to assess the association between DNA oxidative damage and risk of colorectal cancer [5]. The results show that the levels of urinary 8-oxodG were significantly higher in patients than healthy volunteers. Moreover, for patients with colorectal cancer, the concentration of urinary 8-oxodG elevated gradually from stage I to IV, and the level of urinary 8-oxodG in patients with tumor metastasis was significantly higher than that in patients without tumor metastasis. Another study involved 56 colorectal cancer patients, 72 healthy controls and 15 benign tumor patients revealed that combination of urinary 8-oxoG, 8-oxodG and 5-hydroxymethyluracil had better diagnostic power than alone [23].

There is no specific symptom during early colorectal cancer, just the increased frequency of defecation, constipation, diarrhea, secret anguish. Whereas these symptoms are not representative, most people just take it as general gastrointestinal inflammation. At present, occult blood trails accompany with colonoscopy can highly increase the early detection rate of early colorectal cancer. However, colonoscopy is not pervasive in health examinations due to its high price and poor subjective feeling. Oxidative damage biomarkers could reflect oxidative stress level during the early stage of colorectal cancer. Therefore, 8-oxodG and 8-oxoG might have great potential to be novel biomarkers for detection of colorectal cancer.

#### Gastric cancer

The etiologies of the gastric cancer are Helicobacter *pylori* (H. *pylori*), geographical environment, dietary habit (including fast eating speed, hot food and salt preserved food), gene and so on. The pathogenesis of many gastric diseases is partly attributed to oxidative damage. H. *pylori* infection is one of the main risk factor of the gastric cancer. H. *pylori* induces the inflammation of gastric mucosal barrier, resulting in DNA oxidative damage and accumulation of 8-oxodG. This oxidative damage could be a driving force in the progress from chronic gastritis to gastric cancer [24]. In a recent review, it revealed that oxidative DNA damage to stem cells might play an important role in H. *pylori* infection-related gastric cancer through chronic inflammation [25]. This indicates that 8-oxodG might have intimate relationship with occurrence of gastric cancer.

Ni *et al.* detected 8-oxodG and DNA repair enzymes 8-oxoguanine glycosylase (HOGG1) and Mn-superoxide dismutase (MnSOD) in human gastric mucosa with chronic atrophic gastritis (CAG), gastric cancer (GC) and healthy controls. Compared to the controls, 8-oxodG increased in CAG and GC, but HOGG1 reversed [26]. These data support the hypothesis that 8-oxodG may serve as a critical risk factor for the occurrence and development of gastric cancer. We should be careful the elevated levels of 8-oxodG in CAG patients and prevent the potentially occurrence of cancer. Kauppi *et al.* demonstrated that ROS was elevated in the proximal stomach of Barrett’s esophagus and adenocarcinoma patients, which stimulates the proximal stomach mucosa, contributing to the development of cancer in the end [27]. Ma *et al.* found that the level of 8-oxodG in gastric cancer patients was sharply higher than that in normal [28]. All of these results indicate that assessing 8-oxodG level may play an important role in early detection and prevention of gastric carcinoma.

Interestingly, it was found that the exogenous 8-oxodG can strangely reduce ROS production, which could be a potential candidate for the treatment of oxygen-radical-mediated gastric diseases and prevention of oxygen-radical-mediated gastric cancer [29]. However, Dincer *et al.* found that the level of serum 8-oxodG and activity of glutathione were decreased, and superoxide dismutase activity was increased in 19 gastric cancer patients [30]. This results may imply the deficient repair mechanism in cancer patients. Meanwhile, we should get the point that the molecular mechanism of ROS in gastric cancer is still indistinct, and further study still need to be explored.

#### Breast cancer

Breast cancer is one of the prevalent cancers in women and known as an estrogens-dependent disease. Oxidative stress is thought to be an important reason for etiology of kinds of cancers, and breast cancer is no exception. Even under normal physical conditions, the damage to DNA is extensive, let alone oxidative stress [31].

In a study involved 150 both pre- and post-operative breast cancer patients, and 150 female controls, urinary 8-oxodG and other nucleic damage products were sharply higher in pre-operative patients than normal controls, and apparently decreased in post-operations [32]. Urinary 8-oxodG level was found to be higher in the breast cancer patients than that in matched controls [33]. Recently, we have revealed that patients with breast benign lesions could be distinguished from patients with early-stage breast cancer by detection of 8-oxodG in urine [34].

In Sova’s study, they analyzed 8-oxodG in serum and tissue from 173 breast cancer patients. Interestingly, the results shown that low 8-oxodG levels both in serum and immunohistochemical expression were associated with an aggressive breast cancer phenotype, especially in ductal carcinomas, and negative 8-oxodG immunostaining was a powerful prognostic factor in breast carcinoma patients [35]. In addition, other research shown that oxidative stress might have less impact in the pathogenesis of triple-negative breast cancer, compared with other types of breast cancers [36]. Nevertheless, DNA oxidative damage biomarker might still be a promising clinical biomarker in the prediction and prognosis for breast cancer patients.

#### Other cancers

Oxidative damage also has a clear correlation with the irritation and development of other types of cancer. As we all know, the risk factors of lung cancer are the number of cigarettes per day and the persistent time of smoking. Some substances of smoke induce lung oxidative stress damage, eventually turning into cancer [37]. Shen *et al.* demonstrated that patients with higher levels of 8-oxodG had shorter survival time and an over 3-fold increased hazard of death than patients with lower levels [38]. Elevated 8-oxodG in serum or urine was also observed in epithelial ovarian carcinoma (EOC) [39] and esophageal squamous cell carcinoma (ESCC), respectively [40].

### Oxidative damage biomarkers and neurodegenerative disorders

Due to the high energy requirements, high oxygen consumption, and less effective antioxidant systems characteristics, central nervous system can be easily damaged by accumulated ROS [13]. Oxidative nucleic acids damage especially RNA was confirmed closely in touch with the neurodegenerative disorders, such as Alzheimer’s disease (AD), Parkinson disease (PD), epilepsy, prion diseases and so on [10]. Oxidized RNA was observed at the earliest-stage of neurological diseases, and promotes the onset and progress of the lesions, which verified by more and more researches on either human or animal models [41].

### Alzheimer’s disease

AD is one of the most common forms of dementia characterized with extracellular senile plaques composed by amyloid-beta. However the concrete etiology of AD is still unclear, oxidative stress might be a crucial contributor to this disease.

In 1999, Nunomura *et al.* detected the level of 8-oxoG in cytoplasm and nucleoli of neurons of AD patients and controls. Results showed that its levels were higher in the postmortem brain of AD patients than that of controls [42]. Elevated levels of 8-oxoG were observed in hippocampus from patients with AD [43]. Same result was also found in hippocampus of postmortem brain tissue with AD [44]. 8-oxoG concentrations were highly increased in cerebrospinal fluid (CSF) of AD patients [45–46]. Oxidative damage to nucleic acids was identified in neuron of patients with AD from these studies. 8-oxoG is one of the representative RNA oxidative damage biomarker which can reflect the degree of nerve cells oxidative damage on the other side. The more quantity of the biomarkers be detected, the worse state of the illness.

In the study of oxidative m-RNA damage in AD patients, m-RNA was heavily oxidized in frontal cortex that normally serves critical physiological functions [47]. And these were found in mild and moderate stage, not end-stage in AD, suggesting that oxidative damage may be a risk factor in the pathogenesis of AD. Ribosomal RNA is one of the abundant molecules in most cells and is affected readily by ROS in human brain. Ding *et al.* found that oxidized free 5S rRNA might involve in the pathogeny of AD [48]. Wang *et al.* observed the levels of 8-oxoG in mitochondrial were almost 10-fold higher than in nuclear, suggesting that oxidative damage may serve as an etiology of AD [49].

On the whole, oxidative nucleic acids damage might play a significant role in the development of AD and the level of 8-oxoG increased in patients. If these oxidative biomarkers could be detected sensitively at the beginning of AD, we could take some appropriate treatments (medicine therapy, rehabilitation treatment, and good nursing) to delay further deterioration and progression.

### Parkinson’s disease

Parkinson’s disease (PD) is also called shaking palsy, a neurodegenerative movement disorder, pathophysiological characterized with Lewy body and selective loss of dopamine neurons, clinical characterized with muscular rigidity, resting tremor, bradykinesia, and postural instability [50].

The level of 8-oxoG in CSF of patients with PD was much higher than that in controls. For patients, 8-oxoG concentration in CSF was highest in the early stage, reduced sharply with the progress of disease [51]. From this, we can get known the possible role of oxidative RNA damage in the occurrence of early stage of PD. Elevated values of 8-oxodG/8-oxoG are commonly observed in CSF of patients with PD and its levels were much higher in female than male patients [52]. This finding *in vivo* might give us some clues that why PD diseases progresses is faster in females than males. The 8-oxodG concentration was also increased in urine and serum of 6-hydroxydopamine-lesioned rats (PD model) [53]. There also need some further study to determine how the oxidative nucleic acids damage contributes to the onset and progress of PD. The present investigations show that the level of 8-oxoG increased in the early stage of PD and it could be a preferable clinic biochemical biomarker in detection of PD.

### Epilepsy

Epilepsy is a clinical syndrome characterized with multiply neuron abnormal discharge in highly synchronized way. The pathogenesis mechanism of epilepsy is extremely complicated without completely understanding until now. It afflicts more than 50 million people around the world [13]. The specific etiology and mechanism of epilepsy is still unclear, chronic oxidative stress could cause mitochondrial dysfunction and might play a great role in the pathogenesis of epilepsy [54].

There are some studies to investigate the role of oxidative RNA damage in epileptogenesis. Oxidized RNA was greatly increased in vulnerable neurons of mouse brain after pilocarpine-induced epileptic model [13]. A review article also demonstrated a growth of mitochondrial oxidative stress and subsequent cell damage in body after persistent seizures [54]. Rumià *et al.* determined several oxidative stress markers in epileptic and non-epileptic humans and found that 8-oxodG concentrations were significantly higher in patients than controls. This was in accordance with the results in animal models, supporting a closely connection between oxidative DNA damage and epilepsy [55]. In a word, a series of results indicate that ROS-mediated damage may be a critical contributing factor to the onset and evolution of seizure-induced neuron degeneration.

### Other neurological disorders

Nucleic acid oxidation damage in neuronal cells was also identified in patients with dementia with Lewy bodies (DLB) [56–57], prion disease [58–59], amyotrophic lateral sclerosis (ALS) [60] and other neurological disorders. Oxidative damage and its products including 8-oxoG were increased in brain tissue with DLB [56–57]. The presence of abnormal protein in prion diseases is connected with a lot loss of antioxidant defense, which contributes to serious neurodegeneration [58]. Several data ascribed the neuron degeneration of prion diseases to oxidative stress, and antioxidant would be a strong potential therapy for these disorders [59]. Oxygen-mediated m-RNA damage occurred in early pre-symptomatic stage was not a consequence of motor dying neurons in patients with ALS, identified that RNA oxidation is an early factor to deteriorate the motor neuron of ALS [60]. What’s more, same results were found in multiple system atrophy patients and depressive patients [52, 61].

Currently, researches on nucleic acids oxidative damage in neurodegenerative diseases are still not enough. The role of nucleotides oxidation still need to be further studied, and oxidation products could be expected to be prophylaxis elements in these diseases.

### Oxidative damage biomarkers and diabetes mellitus

Diabetes mellitus is a metabolic disease characterized with hyperglycaemia accused to defects in insulin or insulin disorders. Sustained high blood glucose and chronic metabolic disorders could lead to other tissues or organs damage, dysfunction even failure, especially eyes, kidneys, cardiovascular, and nervous system. Several studies have shown that oxidative nucleic acid damage, mostly oxidative DNA damage are closely contacted with pre-diabetes and newly diagnosed diabetes.

It has been revealed that serum 8-oxodG level was significantly higher in patients with pre-diabetes and was positively relate to body mass index (BMI) [62]. And the concentration was also increased in lean normoglycemic offspring of patients with Type 2 diabetes mellitus (T2DM) [63]. Oxidative stress plays important roles not only in T2DM but also in the progress of its complications. 8-OxodG was identified as a useful biomarker in micro-vascular, macro-vascular, and keratopathy complications of T2DM patients [64, 65].

### Oxidative damage biomarkers and cardiovascular disease

ROS could stimulate muscle growth, media reform, and cells dysfunction by activating cytokinin protease and transcription factors, which has a direct impact on cardiac structure and function [66]. Many researches had identified that 8-oxodG was related to cardiovascular diseases. The 8-oxodG levels in serum and urine increased in patients with coronary artery disease [67–69] and heart failure [68–70]. With elevated 8-oxodG level in body fluid, the severity of coronary artery stenosis increased and heart failure was deteriorated [70]. Thus, properties of 8-oxodG could effectively reflect the heart function and chronic failure status, helping us to assess the clinic cardiovascular disease severity [68, 70–71].

## INSPIRATION TO DISEASE TREATMENT AND DRUG DEVELOPMENT

As we described above, ROS plays an important role in pathogenesis of various diseases. Majority studies have also provided the possible new strategy to treat diseases by using antioxidants or medicine to stabilize the redox signal system. There always need some specific biomarkers to estimate the effective degree of disease treatment in clinic practice. The change of 8-oxodG or 8-oxoG level is correlated with the oxidative stress in human body, which providing a hot researching spot for medicine development and therapeutic effects.

Among researches of 8-oxodG/8-oxoG represented as oxidative stress level, there are some antioxidants studies [72–77], basic cell trails [78], and more clinical researches. Some studies indicate that antioxidant, just like sesame oil, effectively protected DNA from oxidative damage [72]. Conventional food naturally contains antioxidants and helps to maintain human health and delay disease onset. Khan and his colleagues highlighted the health beneficial effects of the flavonoid antioxidant fisetin which can be found in many fruits and vegetables, such as apple, onion, and so on. It has potential to be a chemopreventive agent against lots of diseases even cancer [73]. Chemopreventive effects of fisetin were identified in several models. However, there also need further in-depth studies to verify the chemopreventive efficacy *in vivo* as well as other antioxidants. In another study, coenzyme Q10 (CoQ), an additional source of antioxidant in diet, could reduce cellular oxidative progress [74]. Chirayu *et al.* found that adding antioxidants and polyunsaturated fatty acids as supplements in the treatment of neuropsychiatric disorders, had some promising results [75]. In addition, a review supported the notion that habitual dietary intervention of polyphenols can reduce the risk of lung cancer [76]. However, high-dose antioxidant supplements generally do no good and may cause harm to patients [77, 79]. All of these researches provide us a new hotspot for prevention and treatment of ROS-mediated diseases.

Several studies show that oxidative nucleic biomarkers are correlated with the drug efficacy. 8-OxodG was viewed as one of metabolic parameters to evaluate the effect of bezafibrate and fenofibrate treatments in diabetes [80]. Tissue 8-oxodG level in diabetes rats was significantly lower by treated with insulin and antioxidant, and serum 8-oxoG level was almost normalized by insulin. The result supports the important role of antioxidant (probucol and vitamin E) treatment in the reduction of oxidative stress for the prevention of diabetic vascular complications [81]. However, there also some debates on the association between the glycemic control and level of 8-oxoG and 8-oxodG.

ROS is intimately related with the occurrence of many diseases, and the oxidative nucleic acids biomarkers are frequently used as important biochemical indicators in preventive medicine research, such as cancer protection [82–83], nervous system protection [84–87], improvement of cardiovascular function [88–89], and so on. Along with the increase in drug protection research, 8-oxodG and 8-oxoG are increasingly becoming the focus of drug research in the field of oxidative damage.

## DETECTION OF OXIDATIVE NUCLEIC ACID DAMAGE BIOMARKERS

Quantitative detection of the oxidative damage biomarkers of nucleic acids are mainly through two ways including enzyme-linked immunosorbent assay (ELISA) and chromatography-based techniques (Supplementary Table 1). Due to the simple handing process, ELISA is the most convenient method and has received widespread use, but lack of specificity results in higher value than their real value [90]. Other detection methods are based on chromatography techniques, e.g., gas chromatography-mass spectrometry (GC-MS) [91], capillary-electrophoresis with electrochemical detection (CE-ECD) [92], high-performance liquid chromatography with electrochemical detection (HPLC-ECD) [93]. HPLC-MS/MS is of great interest due to its high analytic sensitivity, accuracy and reliability, and it is the mainstream solution for quantitative detection of oxidative damage biomarker in human body fluid and tissue [5, 32, 34]. The sample was pretreated by solid phase extraction (SPE), and then the target analytes were separated by HPLC. Finally, these compounds were detected by mass spectrometer under multiple reaction monitoring mode. With the rapid development of these analytical techniques, evaluation of the potential of oxidative nucleic acid damage biomarkers for detection of diseases becomes more accurate and convenient.

## CONCLUSIONS AND PERSPECTIVES

More and more researches have demonstrated that increased oxidative damage on nucleic acids directly or indirectly cause dysfunction and detrimental effects to cells, organs and entire body. As the most prevalent oxidative products, 8-oxodG and 8-oxoG could be utilized to estimate the level of intracellular oxidative damage and reflect the internal environment of sick body. Their levels in biofluids or tissue can provide useful and visual messages about morbidity risk assessment, diseases course and therapy effects. 8-OxodG was increased in many different solid cancers and chronic diseases, and 8-oxoG was elevated in neurodegenerative diseases. As we all know, it is difficult to screen disease by only one biomarker. But if we can combine 8-oxodG or 8-oxoG with traditional testing or imaging method, the diagnosis sensitivity of some diseases would increase. We might get several clues from these oxidative damage biomarkers and they are worth being studied as biomarkers for detection of diseases. Moreover, 8-oxodG/8-oxoG may provide us a new point of view to new drug research and development for these oxidative-stress-mediated diseases. However, occurrence and development of diseases are attributed to multiple factors. Oxidative nucleic acid damage may be one of the links in this program, and other unknown parts still needing further deeply exploration. At present, the clinical application of 8-oxodG/8-oxoG is still in early stage study, and these studies are lack of reasonable prospective design and placebo control. In future, we should conduct more long-term, large-scale randomized control studies, confirming the concrete relationship between 8-oxodG/8-oxoG and the onset, course, development and prognosis of diseases.

## SUPPLEMENTARY MATERIALS TABLE




