# Supplementary material for: Potential application of the oxidative nucleic acid damage biomarkers in detection of diseases
Source: Oncotarget. 2017 Sep 8;8(43):75767–77. doi: 10.18632/oncotarget.20801 (PMC5650463; doi:10.18632/oncotarget.20801)
Supplement: Supplementary file 1 [file oncotarget-08-75767-s001.pdf]

www.impactjournals.com/oncotarget/ Oncotarget Supplementary Materials

Potential application of the oxidative nucleic acid damage biomarkers in detection of diseases

SUPPLEMENTARY MATERIALS

Supplementary Table 1: Summary of studies in oxidative nucleic acid damage biomarkers. See Supplementary_Table_1
