# Supplementary material for: Potential application of the oxidative nucleic acid damage biomarkers in detection of diseases
Source: Oncotarget. 2017 Sep 8;8(43):75767–77. doi: 10.18632/oncotarget.20801 (PMC5650463; doi:10.18632/oncotarget.20801)
Supplement: Supplementary file 2 [file oncotarget-08-75767-s002.doc]

**Supplementary Table 1: Summary of studies in oxidative nucleic acid damage biomarkers.**

| **Diseases** | **Analytical method** | **Sample** | **Results** | **Ref.** |
| --- | --- | --- | --- | --- |
| **Colorectal cancer (CRC)** | HPLC-MS/MS | urine | 8-OxodG: 1.68 ± 0.85 nmol/mmol creatinine in CRC patients versus 1.07 ± 0.49 nmol/mmol creatinine in healthy controls. | 5 |
|  | ELISA | plasma | Mean 8-oxodG level in patients with early cancer was highest | 21 |
|  | ELISA | serum | 8-OxodG: 24.07 ± 5.11 ng/ml in CRC patients versus 5.95 ± 1.83 ng/ml in controls; 8-oxodG levels were 4-fold higher in CRC patients than that in controls. | 22 |
|  | GC-MS | urine | 8-OxodG: 2.47 ± 1.31 nmol/mmol creatinine in CRC patients versus 1.79 ± 0.58 nmol/mmol creatinine in controls; 8-oxoG: 8.25 ± 10.22 nmol/mmol creatinine in CRC patients versus 4.58 ± 3.55 nmol/mmol creatinine in controls. | 23 |
| **Gastric cancer**  **(GC)** | ICC | gastric mucosa | 8-OxodG level in GC patients was higher than control | 26 |
|  | ELISA | serum | 8-OxodG: 16.34 ± 8.30 ng/ml in GC patients versus 12.29 ± 5.72 ng/ml in controls. | 28 |
|  | ELISA | plasma | 8-OxodG: 1.00 ± 0.34 ng/ml in CRC patients versus 3.39 ± 0.75 ng/ml in controls | 30 |
| **Breast cancer**  **(BC)** | LC-MS/MS | urine | 8-OxodG: 0.02±0.01 nmol/μmol creatinine in pre-operations versus 0.01±0.01 nmol/μmol creatinine in post-operations | 32 |
|  | HPLC-ECD | urine | 8-OxodG: 16.39 ± 17.3 μmol/mol creatinine in BC patients versus 4.70 ± 7.1 μmol/mol creatinine in controls | 33 |
|  | HPLC-MS/MS | urine | 8-OxodG: 1.88 ± 1.28 nmol/mmol creatinine in BC patients versus 1.11 ± 0.6 nmol/mmol creatinine in non-cancerous. | 34 |
| **Lung cancer** | ELISA | tissue | Patients with lower level of 8-oxodG had longer survival time | 38 |
| **Epithelial ovarian carcinoma (EOC)** | ELISA | serum | High serum 8-oxodG (>140 pg/ml) was associated with poor ovarian cancer-specific survival | 39 |
| **Esophageal squamous cell carcinoma (ESCC)** | ELISA | urine | 8-OxodG: 15.0 ± 5.1 ng/mg creatinine in SCC patients versus 5.8 ± 2.1 ng/mg creatinine in controls | 40 |
| **Alzheimer’s disease (AD)** | ICC | tissue | 8-OxoG and 8-oxodG significantly increased in patients with AD as compared with controls | 42 |
|  | HPLC-EC-UV | tissue | 8-OxoG particularly increased in RNA and heterogeneously distributed among adjacent regions versus the controls | 43 |
|  | ICC | tissue | RNA oxidative damage (8-oxoG) increased in patients with AD | 44 |
|  | HPLC-ECD | CSF | 8-OxoG: 500 ± 213 pM in AD patients versus 97 ± 32 pM in controls | 46 |
|  | GC-MS | tissue | 8-OxodG in nDNA and mtDNA of different local brain tissue (frontal, parietal, temporal lobes and cerebellum) was higher (nearly 2-fold) in patients with AD than that in controls | 49 |
| **Parkinson’s disease**  **(PD)** | HPLC-ECD | serum, CSF | CSF 8-oxoG: 288 ± 129 pM in PD patient versus 97 ± 32 pM in controls; serum 8-oxoG: 1.49 ± 0.54 pM in PD patients versus 1.42 ± 0.59 pM in controls | 51 |
|  | ELISA | serum, CSF | CSF 8-oxodG and 8-oxoG: 2.85 ± 2.43 ng/ml in PD patients versus 1.46 ± 0.83 ng/ml in controls, serum 8-oxodG and 8-oxoG: 57.5 ± 20.4 ng/ml in PD patients versus 35.7 ± 14.6 ng/ml in controls | 52 |
|  | ELISA | serum, urine | 8-OxodG in rats with 2 days post-6- hydroxydopamine-lesion: urine, 4.3 ± 0.8 ng/mg creatinine (115% increase to sham controls); serum, 9.6 ± 0.4 ng/mg (30% increase to sham controls) | 53 |
| **Epilepsy** | HPLC-ECD | tissue | 8-OxodG levels were nearly 3.5-fold higher in epilepsy patients than that in controls | 55 |
| **Multiple System Atrophy (MSA)** | ELISA | serum, CSF | CSF 8-oxodG and 8-oxoG value significantly increased in MSA (4.24 ± 3.53 ng/ml) | 52 |
| **Dementia with Lewy bodies (DLB)** | GC-MS | tissue | Oxidized DNA bases increased in DLB patients than that in controls | 57 |
| **Amyotrophic lateral sclerosis (ALS)** | IP, SB, RT-PCR | tissue | mRNA oxidation increased in ALS postmortem tissue, and in transgenic mice at early pre-symptomatic stage | 60 |
| **Diabetes** | ELISA | serum | 8-OxodG: 671.3 ± 140 pg/ml in prediabetic versus 210.1 ± 166 pg/ml in control, highest 8-oxodG values (1979.6 ± 1209 pg/ml) in diabetic group | 62 |
|  | ELISA | serum | 8-OxodG increased in lean normoglycemic offspring of T2DM, 0.245 ± 0.715 ng/ml versus 0.126 ± 0.238 ng/ml in control | 63 |
|  | ELISA | urine | 8-OxodG increased in T2DM and its complications | 64 |
| **Cardiovascular diseases** | ELISA | serum | 8-OxodG: 0.41 (0.30-0.57) ng/ml in patients with coronary artery disease versus 0.32 (0.25-0.43) ng/ml in patients with normal coronary artery | 67 |
|  | ELISA | urine | 8-OxodG level increased in coronary artery diseases and heart failure | 68 |
|  | ELISA | serum, urine | In chronic heart failure patients, 8-oxodG in coronary sinus (0.56 ± 0.46 ng/ml) was higher than that in the aortic root (0.27 ± 0.14 ng/ml). Urine 8-oxodG: 11.9 ± 2.0 ng/mg versus 8.0 ± 2.3 ng/mg in controls | 70 |

**ELISA** enzyme-linked immunosorbent assay, **HPLC** high-performance liquid chromatography, **GC** gas chromatography, **ICC** immunocytochemistry, **MS** mass spectrometry, **ECD** electrochemical detection, **UV** ultraviolet, **IP** immunoprecipitation, **RT-PCR** reverse transcription polymerase chain reaction, **SB** Southern-Blot.
